# The direct and indirect effects of lurasidone monotherapy on functional improvement among patients with bipolar depression: results from a randomized placebo-controlled trial

**DOI:** 10.1186/s40345-016-0049-1

**Published:** 2016-03-16

**Authors:** Krithika Rajagopalan, Elizabeth Dansie Bacci, Kathleen W. Wyrwich, Andrei Pikalov, Antony Loebel

**Affiliations:** Sunovion Pharmaceuticals Inc., Marlborough, MA USA; Evidera, Bethesda, MD USA; Sunovion Pharmaceuticals Inc., Fort Lee, NJ USA; Evidera, 1417 Fourth Avenue, Suite 510, Seattle, WA 98101 USA

**Keywords:** Sheehan disability scale, Patient functioning, Bipolar disorder, Depression

## Abstract

**Background:**

Bipolar depression is characterized by depressive symptoms and impairment in many areas of functioning, including work, family, and social life. The objective of this study was to assess the independent, direct effect of lurasidone treatment on functioning improvement, and examine the indirect effect of lurasidone treatment on functioning improvement, mediated through improvements in depression symptoms.

**Methods:**

Data from a 6-week placebo-controlled trial assessing the effect of lurasidone monotherapy versus placebo in patients with bipolar depression was used. Patient functioning was measured using the Sheehan disability scale (SDS). Descriptive statistics were used to assess the effect of lurasidone on improvement on the SDS total and domain scores (work/school, social, and family life), as well as number of days lost and unproductive due to symptoms. Path analyses evaluated the total effect (β_1_), as well as the indirect effect (β_2_×β_3_) and direct effect (β_4_) of lurasidone treatment on SDS total score change, using standardized beta path coefficients and baseline scores as covariates. The direct effect of treatment on SDS total score change and indirect effects accounting for mediation through depression improvement were examined for statistical significance and magnitude using MPlus.

**Results:**

In this 6-week trial (*N* = 485), change scores from baseline to 6-weeks were significantly larger for both lurasidone treatment dosage groups versus placebo on the SDS total and all three SDS domain scores (*p* < 0.05). Through path analyses, lurasidone treatment predicted improvement in depression (β_2_ = −0.33, *p* = 0.009), subsequently predicting improvement in functional impairment (β_3_ = 0.70, *p* < 0.001; indirect effect = −0.23). The direct effect was of medium magnitude (β_4_ = −0.17, *p* = 0.04), indicating lurasidone had a significant and direct effect on improvement in functional impairment, after accounting for depression improvement.

**Conclusions:**

Results demonstrated statistically significant improvement in functioning among patients on lurasidone monotherapy compared to placebo. Improvement in functioning among patients on lurasidone was largely mediated through a reduction in depression symptoms, but lurasidone also had a medium and statistically significant independent direct effect in improving functioning.

## Background

Bipolar disorder is a chronic and debilitating mental illness characterized by recurrent episodes of hypomania, mania, and depressive symptoms (Goodwin and Jamison 2007). It is ranked by the World Health Organization as one of the top 20 causes of disability worldwide (Vos et al. 2012). The symptoms of bipolar disorder may often result in serious functional impairment and quality of life declines (Henry et al. 2013), primarily due to its early onset (approximately 18 years) and chronic nature.

In short-term clinical trials for the treatment of bipolar depression, the primary focus has traditionally focused on the alleviation of symptoms of bipolar depression. Remission of depressive symptoms is typically measured from clinician-reported outcome assessments of depression, such as a Montgomery–Asberg depression rating scale (MADRS) score ≤10 (Montgomery and Asberg 1979) or a Hamilton rating scale for depression (HAM-D) score ≤7 (Hamilton 1967). Since clinician-reported measures often do not capture treatment impact on functional status, activities of daily living, or quality of life, all of which are areas that are important in the context of functional impairment for patients (Kessler et al. 2006; Miklowitz 2011; Harvey 2011; Greer et al. 2010), recent emphasis has been placed on patient-reported outcome measures to complement symptomatic assessments in this population (Rosa et al. 2010). Indeed, some argue that patient-reported measures of psychosocial functioning that reflect how a patient feels or functions are more meaningful outcomes than clinician-reported measures of symptomatic remission (Keck 2004).

Given the important role of functional and quality of life parameters in determining the long-term effectiveness of treatment, an understanding of the direct and indirect effect of drug treatment on functional improvement is vital. Thus, this post hoc analysis of a placebo-controlled monotherapy trial of lurasidone treatment among patients with bipolar depression was conducted to (1) describe the effect of lurasidone treatment on improvement in functional impairment (as measured by the SDS); and (2) examine the direct effect of lurasidone treatment on functional improvement, and assess the indirect effect (mediated through improvement in bipolar depression symptoms) of lurasidone in improving patient functioning.

## Methods

### Study design and data source

The 6-week, multicenter, randomized, double-blind, placebo-controlled, parallel-group bipolar depression monotherapy trial of lurasidone was the data source for this analysis (NCT00868699). This trial has been described in detail in a previous publication (Loebel et al. 2014b). Patients that completed the study at 6-weeks from a total of 505 randomized subjects receiving at least one dose of study medication at doses of 20–60 or 80–120 mg/day, with at least one baseline measurement of the MADRS and one post-baseline efficacy measurement for the MADRS formed the analytical sample (defined as the completer population). All patients were 18–75 years of age, experiencing a major depressive disorder (DSM-IV-TR criteria, ≥4 weeks and <12 months in duration), with or without rapid cycling, without psychotic features, and with a history of at least one lifetime bipolar manic or mixed manic episode. The DSM-IV-TR diagnosis was confirmed via an interviewer-administered structured interview (Mini-International Neuropsychiatric Interview).

The study was approved by an institutional review board at each investigational site and was conducted in accordance with the International Conference on Harmonisation of Good Clinical Practices guidelines and with the ethical principles of the Declaration of Helsinki.

## Scales and assessments included in the analysis

### Symptom assessment

#### Montgomery–Asberg depression rating scale (MADRS)

The patient’s primary bipolar depression symptoms were assessed using the MADRS, a clinician-administered rating scale developed from a larger scale to be sensitive to change (Montgomery and Asberg 1979). The MADRS has ten items, each scored on a 0–6 scale. A score of zero indicates an absence of that symptom, and anchor point descriptors are given for sores of 0, 2, 4, and 6. Items assess many facets of depression, including sadness, tension, pessimism, suicidal thoughts, reduced sleep, and reduced appetite. Higher scores indicate greater depression severity with a maximum total score of 60.

### Functional impairment assessment

#### Sheehan disability scale (SDS)

Functional impairment was assessed using the Sheehan disability scale (SDS) (Sheehan et al. 1996; Sheehan and Sheehan 2008), a measure validated for use in patients with bipolar disorder (Arbuckle et al. 2009). The SDS is a composite of three self-rated items designed to measure the extent to which three major sectors in the subject’s life are impaired by panic, anxiety, phobic, or depressive symptoms: work/school, social life, and family life. The three items are rated using an 11-point visual analog scale ranging from 0 to 10. In addition, two questions are used to assess the number of days lost due to symptoms and the number of days unproductive due to symptoms over the last week. The SDS total score is the sum of the three items and ranges from 0 (unimpaired) to 30 (highly impaired).

### Additional measures to characterize the patient population

In addition to the primary symptom and functional impairment measures, three additional scales were also used to characterize the patient population. The Hamilton Anxiety Rating Scale (HAM-A) (Hamilton 1959) is a 14-item clinician-rated assessment to measure the severity of anxiety symptoms. Each item is rated on a 5-point scale from 0 (not present) to 4 (severe/disabling), with a total score range of 0–56. The quality of life satisfaction and enjoyment-short form (Q-LES-Q SF) is a 16-item health-related quality of life measure of the degree of enjoyment and satisfaction experienced by patients in various areas of daily living (Endicott et al. 1993). The 16 items reduce to eight summary scales that reflect major areas of functioning: physical health, mood, leisure time activities, social relationships, general activities, work, household duties, and school/coursework. Each item is rated on a 5-point scale, ranging from 1 (very poor) to 5 (very good). The sum of scores for items 1–14 can range from 14 to 70, and is expressed as a percentage (0–100) of the maximum total score that is achievable (items 15 and 16 were not used in the present analyses). Finally, the 16-Item quick inventory of depressive symptomology (QIDS-SR_16_) is a 16-item patient-reported outcome measure of depressive symptomology that converts the responses to 16 items into the 9 DSM-IV symptom criterion domains. Each item is rated on a 4-point scale, with higher scores indicative of greater symptomology (total score range of 0–27) (Rush et al. 2003).

## Statistical methods

### Descriptive statistics

The demographic and baseline characteristics of all patients were summarized using descriptive statistics (mean, standard deviation, range, frequencies for categorical variables). In the clinical trial, efficacy assessments were obtained at baseline and at weekly intervals, for up to 6-weeks, with the primary efficacy endpoint being a mean change in MADRS total score (∆MADRS) from baseline to 6-weeks (Loebel et al. 2014b). For the current analysis, mean change in SDS total score (∆SDS total), individual domain scores (work/school, family life, social life), and the number of days lost and number of days unproductive from baseline to 6-weeks was used to describe the efficacy of treatment on improvement in functional impairment. Independent samples *t*-tests were conducted to assess for statistically significant differences in mean change scores between the lurasidone treatment groups versus placebo.

### Path analysis

Path analysis was conducted (collapsing across dosage groups) to assess the relationship between treatment and ∆SDS total score directly and through ∆MADRS and to quantify the total (direct and indirect) effects of treatment on improvement in functional impairment at 6-weeks, following the procedures described by Baron and Kenny (Baron and Kenny 1986). The Baron and Kenny mediation model was used to assess the degree of the treatment effect upon a response variable in the presence of another variable (i.e., the mediating variable). This approach allowed the examination of the degree of mediation (either as partial or complete mediation), through a series of four models. Statistically significant (*p* < 0.05) effects must be obtained in Models 1, 2, and 3 in order to test the full mediation model in Model 4. Specifically, ∆MADRS would be considered a partial mediator if: (Model 1) treatment significantly predicts ∆SDS total; (Model 2) treatment significantly predicts ∆MADRS; (Model 3) independent of treatment, ∆MADRS linearly significantly predicts ∆SDS total; and (Model 4) treatment significantly predicts ∆SDS total, even when controlling for the effect of ∆MADRS. Complete mediation would be indicated if the effect of treatment on SDS described in Model 4 was 0. Each of the models controlled for baseline score of the dependent variable (i.e., SDS total or MADRS).

Standardized parameter estimates with corresponding p-values were calculated for all four models. The total effect (β_1_) of the relationship between treatment and ∆SDS total is estimated in Model 1. The direct effect of treatment on the ∆SDS total controlling for ∆MADRS (β_4_), and the indirect effect, was estimated in Model 4. The indirect effect was calculated as the product of the relationship between treatment and ∆MADRS (β_2_), and the relationship between ∆MADRS and ∆SDS total (β_3_). The proportion of the effect that is mediated was calculated as β_2_×β_3_/β_1_, while the percentage of total variance explained by each path was reported using the standardized *R*^2^ values.

The strength of the parameter estimates was interpreted using Kenny’s recommendations for estimates of small (0.02), medium (0.15), and large (0.40) (Kenny  2014) effect sizes. Overall model fit was assessed using various global fit indices, where the following indices and fit values were used as criteria to assess acceptable model fit: Chi-square test of overall model fit; root mean square error of approximation (RMSEA) <0.06 (MacCallum et al. 1996); and Tucker–Lewis index (TLI) and the comparative fit index (CFI) >0.90 (Hu and Bentler 1999). Mplus statistical software version 7.0 (Muthén and Muthén 1998–2013) was used to conduct all mediation analyses.

## Results

### Demographics and baseline characteristics

A total of 818 patients were screened, of whom 505 (61.7 %) were randomly assigned to 6-weeks of treatment and 280 (55.4 %) were included in the completer study population. Baseline demographic characteristics were similar for treatment (20–60 and 80–120 mg/day) and placebo groups, in addition to clinical characteristics, including clinician- and patient-completed assessments (Table 1). More extensive patient baseline characteristics can be found in Loebel and colleagues (Loebel et al. 2014b).

**Table 1:** Patient demographic characteristics of completer population at baseline (*N* = 280)

	Lurasidone 20–60 mg/day (*N* = 90)	Lurasidone 80–120 mg/day (*N* = 96)	Placebo (*N* = 94)
Age, mean (SD)	41.2 (12.9)	41.7 (12.6)	39.1 (11.4)
Male, *n* (%)	43 (47.8 %)	41 (42.7 %)	44 (46.8 %)
White, *n* (%)	60 (66.7 %)	62 (64.6 %)	62 (66.0 %)
SDS total score, mean (SD)	10.1 (7.3)	10.0 (7.4)	13.3 (8.3)
MADRS total score, mean (SD)	29.9 (4.7)	29.7 (4.8)	29.8 (4.7)
HAM-A total score, mean (SD)	16.2 (6.6)	14.9 (4.9)	15.6 (6.1)
Q-LES-Q SF total score, mean (SD)	33.4 (13.8)	34.6 (12.8)	36.5 (13.2)
QIDS-SR_16_ total score, mean (SD)	14.0 (3.7)	14.3 (3.1)	14.5 (3.2)

### Changes in patient functioning outcomes

#### Descriptive statistics

**Fig. 1:**
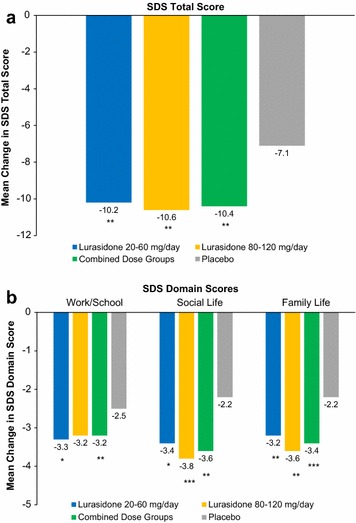
Mean change for SDS total (**a**) and domain (**b**) scores from baseline to 6-weeks by treatment group. Lurasidone vs. Placebo: *p < 0.05; **p < 0.01; ***p < 0.001; ****p < 0.0001

Mean change scores for the SDS total and domain scores from baseline to 6-weeks are presented in Fig. 1. Change scores were significantly larger for both lurasidone dosage groups versus placebo on the SDS total and all three SDS domain scores. In addition, patients on lurasidone in both dosage groups and the combined dosage group reported a significantly fewer number of days lost and less unproductive days. Specifically, patients receiving lurasidone reported a mean change of −1.3 (SD = 2.2), −1.8 (SD = 2.4), and −1.6 (SD = 2.3) number of days lost for the lurasidone 20–60, 80–120 mg/day, and combined lurasidone treatment groups in comparison to −1.1 (SD = 2.4) for placebo (all *p* < 0.001). Similarly, patients in all lurasidone treatment groups reported a mean change of −2.5 (SD = 2.8) days unproductive in comparison to −1.1 (SD = 2.7) for placebo (all *p* < 0.001).

### Direct and indirect effects of lurasidone on improvement in functional impairment

Relationships tested in Models 1, 2, and 3 were statistically significant, thus, the full mediation model (Model 4) was analyzed. Specifically, path analysis of Model 1 revealed a moderate total effect for lurasidone treatment predicting improvement in the SDS total score (β_1_ = −0.40, *p* = 0.001). Similarly, Models 2 and 3 demonstrated moderate effects for the relationships between lurasidone treatment and improvement in MADRS (β = −0.41; *p* = 0.001), as well as improvement in MADRS on improvement in the SDS total score (β = 0.70; *p* < 0.001).

**Fig. 2:**
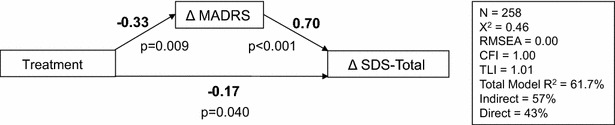
Full Mediation Model. *RMSEA* root mean square error of approximation; *CFI* comparative fit index; *TLI* Tucker–Lewis index; *Indirect,* percentage of change in SDS variance explained by indirect effects; *Direct,* percentage of change in SDS variance explained by direct effect

As shown in Fig. 2 depicting Model 4, treatment predicted improvement in MADRS (β_2_ = −0.33, *p* = 0.009), which subsequently predicted improvement in the SDS total score (β_3_ = 0.70, *p* < 0.001; indirect effect = −0.23). The direct effect was of medium magnitude and significant (β_4_ = −0.17, *p* = 0.04), indicating partial mediation. Indirect and direct effects accounted for 57 and 43 % of the total effect, respectively. The full mediation model with indirect and direct effects explained 61.7 % of the variation in the change in the SDS total score.

## Discussion

After 6-weeks, monotherapy lurasidone patients at a dose range of 20–120 mg/day performed significantly better and had greater reduction in functional impairment, as indicated by the SDS total score, compared to those on placebo. Similar change from baseline to 6-weeks was demonstrated across each of the SDS domain scores (work/school, family, and social life), indicating that all three domains contributed equally to the SDS total score improvement. Further, patients receiving all doses of lurasidone treatment also reported significantly fewer days lost and days unproductive due to symptoms in comparison to placebo. These domain-specific findings demonstrate that at various dosage levels, lurasidone effectively improved functional impairment in patients with bipolar disorder in all areas of impairment assessed.

Previous research has demonstrated that lurasidone significantly improved symptoms of bipolar depression and functional impairment (Loebel et al. 2014a, b). The current analysis extends these findings by assessing if improvement in functional impairment (SDS) due to lurasidone treatment was independent of improvement in bipolar depression symptoms (MADRS). Path analysis revealed that improvement in SDS total scores was largely but not completely explained by improvement in MADRS. The remaining statistically significant and medium-strength direct effect (β = −0.17), after accounting for the indirect effect of MADRS change on SDS total score change (57 % of the total effect), revealed that treatment had an independent effect on improvement in functional impairment.

These findings are supported by a recent post hoc analysis of the original 485 patients included in the lurasidone efficacy analyses reported by Loebel and colleagues (Loebel et al. 2014b). In this post hoc analysis, Loebel and colleagues estimated rates of recovery, defined by combined symptomatic remission (MADRS ≤12) and functional remission (all SDS domain scores ≤3) sustained for at least 3 months in the 6-month continuation study, in patients treated with lurasidone monotherapy (Loebel et al. 2015). The proportion of lurasidone-treated patients attaining symptomatic remission (defined as a MADRS total score ≤12) at week 6 was significantly higher (40.9 %) compared to placebo (24.7 %, *p* < 0.01) (Loebel et al. 2015). Similarly, the proportion of lurasidone-treated patients achieving functional remission at week 6 was significantly higher (48.4 %) compared to placebo (31.5 %, *p* < 0.01). However, as the current analysis utilized path analysis to assess for direct and indirect effects of treatment, it is impossible to rule out potential pseudo-specific effects on the patient’s report of functioning improvement.

The clinical importance of these findings is supported by a body of research that has documented the value of assessing functional impairment as a disability endpoint in this population. Multiple investigations have demonstrated that patients with bipolar depression are significantly more likely to report impairment in functional areas valued by patients, including relationships with family and friends, functioning at work and school, and cognitive impairment (Keck 2004; Rosa et al. 2010; Altshuler et al. 2006; Tohen et al. 2000; Simon et al. 2007; Henry et al. 2013; Depp et al. 2012; Gutierrez–Rojas et al. 2011; Calabrese et al. 2004). Further, even patients in remission from depressive symptoms may show continued, impaired psychosocial functioning (Rosa et al. 2010; Greer et al. 2010), demonstrating the need to assess functional impairment even in the absence of continued depressive symptoms. Thus, patient-centered assessment tools such as the SDS are valuable for measuring change in functional and disability outcomes important to patients with bipolar disorder that may not be captured using clinician-completed symptom assessments such as the MADRS. Our findings support the need for independent assessment patient functional improvement as an endpoint when assessing the efficacy of antipsychotic or antidepressant treatment. However, it should be noted that although the SDS was developed for the assessment of functional impairment in clinical trials of depression, anxiety, and bipolar disorders, the SDS is often found to be moderately correlated with measures of depression. Indeed, in the present study, the SDS Total score was found to be moderately correlated with the MADRS (*r* = 0.37). Thus, the findings of the present study are preliminary, as no clinician- or performance-based assessment of functional impairment was available to use to confirm the patient’s report of improvement in functioning specifically.

Although treatment remained a statistically significant predictor of improvement in functional impairment, changes in bipolar depression symptoms accounted for the majority of the effect on change in functional impairment. As has been elaborated by the FDA PRO guidance document (Food and Drug Administration 2009), a well-validated PRO should be able to measure the effects of a treatment on “how a patient feels or functions” both through the direct and mediated effect on symptom improvements. The findings of this analysis are consistent with previous work that has demonstrated that mood symptoms (such as depression) may actually be independent predictors of functioning in patients with bipolar depression (Burdick et al. 2010) or major depressive disorder (Sheehan et al. 2011; Wise et al. 2008). For example, Sheehan and colleagues (Sheehan et al. 2011), using the SDS to assess functional outcomes, conducted multivariate lead-lag regression analyses to demonstrate that changes in mood and depressive symptoms at weeks 6 and 7 (during an episode or in a treatment trial) were significantly correlated with changes in the SDS total score at week 8, indicating that changes in mood symptoms associated with treatment significantly predicted change in functional outcomes. Using a causal modeling approach in the current study, our findings supported those of Sheehan and colleagues, as improvement in symptoms of bipolar depression, in turn, resulted in improvement in functional impairment.

The findings from the mediation analysis have particular clinical importance as it relates to the treatment of individuals with bipolar depression. Specifically, it is possible to reduce disability and functional impairment, in conjunction with symptomatic remission with the same treatment. Indeed, Simon and colleagues (Simon et al. 2007) conducted a secondary analysis of a 12-month randomized trial of a care management and psychoeducational intervention for bipolar disorder. These researchers found that within-person improvement in depression severity due to treatment was associated with clinically significant improvement in impairment and disability. While some researchers advocate for interventions that specifically target improvement in functional outcomes (Rosa et al. 2010), our findings provide preliminary evidence for an efficient treatment that has been demonstrated to improve function, both mediating through and independently from a reduction in depressive symptoms. However, further research using a causal study design is needed to confirm these findings.

Several study limitations should be noted. First, this study was a post hoc analysis of a short-term acute trial. A longitudinal study is needed to confirm that patients maintained these improvements over a longer period of time, and to determine if the mediating effect of depressive symptom severity improvement on reduced functional impairment persists. Second, functional impairment was assessed based on patient-reported data of patients that had not yet achieved remission status, and not through direct observation of patient behavior and functioning. Similar to many registration clinical trials facing limited resource issues, functional impairment was measured by the SDS as one of the secondary endpoints, and our clinical trial did not employ additional informant assessment such as clinician-reported measures of functioning or performance-based assessments to validate the self-reported functioning outcomes. Along the same lines, no neuropsychological measures were administered to further understand the psychological functioning of the patient population at baseline or study completion. Thus, our analyses and the conclusions that can be drawn in our study are exploratory in nature and need further confirmation from future investigations. Finally, in regards to the path analysis, our sample size was limited and our tested models should be re-estimated in a larger population, whenever possible in future studies. While mediation analyses allow for the interpretation of a causal association between constructs, these findings are preliminary and further analysis using a causal study design will help to substantiate the current findings that changes in bipolar depression were causally associated with changes in functional impairment.

## Conclusions

In conclusion, the findings from the present study indicate that lurasidone as a monotherapy is efficacious in improving functional impairment in patients with bipolar depression, in addition to reducing depressive symptoms. Lurasidone largely improved functional impairment indirectly through reductions in depressive symptoms, with a smaller effect evidenced directly between treatment and improved functional impairment. These findings underscore the need for patient-reported outcomes to complement clinician-reported measures in understanding the value of treatment for patients. This analysis further illustrates the importance of treatment selection in addressing patient-centered issues relating to symptomatology and functional improvement in chronic mental illnesses such as bipolar disorder.

## Abbreviations

CFI: comparative fit index
HAM-A: Hamilton anxiety rating scale
HAM-D: Hamilton rating scale for depression
MADRS: Montgomery–Asberg depression rating scale
∆MADRS: mean change in MADRS total score
QIDS-SR_16_: 16-item quick inventory of depressive symptomology
Q-LES-Q SF: quality of life satisfaction and enjoyment-short form
RMSEA: root mean square error of approximation
SDS: Sheehan disability scale
∆SDS: total mean change in SDS total score
TLI: Tucker–Lewis index
